# Exercise as a modulator of purinergic signaling and inflammation in cancer-associated sarcopenia: a narrative review

**DOI:** 10.1007/s00011-026-02331-5

**Published:** 2026-09-05

**Authors:** Emeline Moraes de Oliveira, Vitor Mariano Gonçalves Dockhorn, João Victor Garcia de Souza, Francini Franscescon, Débora Tavares de Resende e Silva

**Affiliations:** 1 Medicine Department, Federal University of Fronteira Sul (UFFS), Chapecó, Santa Catarina Brazil; 2 Graduate Program in Biomedical Sciences, Federal University of Fronteira Sul (UFFS), Rodovia SC 484 - Km 02, Fronteira Sul, Chapecó, Santa Catarina CEP 89815-899 Brazil

**Keywords:** Physical exercise, Sarcopenia, Purinergic system, Inflammation, Cancer

## Abstract

**Background:**

Sarcopenia, characterized by the progressive loss of skeletal muscle mass and strength, is highly prevalent among cancer patients and is strongly associated with poor prognosis. Increasing evidence indicates that overactivation of the purinergic system contributes to a chronic inflammatory state that promotes tumor progression and exacerbates muscle wasting.

**Purpose:**

This narrative review examines how physical exercise may modulate purinergic signaling and inflammation, potentially improving the quality of life of cancer patients with sarcopenia.

**Methods:**

A literature search was conducted in the MEDLINE database via PubMed, including articles published between 2008 and 2025, using descriptors such as “purinergic signaling”, “CD39”, “CD73”, “P2X7 receptor”, “adenosine”, “sarcopenia”, “skeletal muscle metabolism”, “cancer”, “inflammation”, and “extracellular ATP”.

**Results:**

Original studies and review articles in English addressing the relationship between purinergic pathways, inflammation, muscle metabolism, and cancer were included.

**Conclusions:**

Findings suggest that cancer-related sarcopenia is closely linked to systemic inflammation mediated by purinergic pathways, and evidence indicates that exercise may play a pivotal role in mitigating muscle loss and improving patient outcomes.

## Introduction

Sarcopenia, a condition characterized by progressive loss of skeletal muscle mass and decreased strength, has a variable incidence depending on the diagnostic criteria used, but it is widely recognized as a prevalent problem in aging. In cancer patients, this condition takes on greater clinical relevance, as it is associated with a worse prognosis and negative outcomes throughout treatment [1]. The prevalence of sarcopenia in oncological populations depends on the diagnostic method used, but its impacts are widely recognized: loss of functionality, worsening quality of life, longer hospital stays, and reduced survival [2]. Among the main factors associated with sarcopenia in individuals with cancer are reduced physical activity and the side effects of chemotherapy on muscle tissue [3]. Some studies suggest the importance of early assessment of muscle mass loss, both before and after chemotherapy treatment, as a strategy for risk stratification and appropriate clinical management [4].

Physical inactivity, frequently present in cancer patients, is an aggravating factor. Since muscle tissue functions as an important energy reservoir in situations of metabolic stress such as neoplasms and chemotherapy, individuals with reduced physical reserves or greater vulnerability may experience more unfavorable clinical outcomes [5]. In this context, regular physical exercise emerges as a promising non-pharmacological strategy for both the prevention and treatment of sarcopenia in cancer patients [6]. The combination of resistance exercises and aerobic activities has proven effective in promoting muscle hypertrophy, maintaining lean mass, and improving muscle function, in addition to contributing to metabolic and cardiovascular health [7]. Therefore, implementing supervised physical exercise programs with training adapted to individual conditions and limitations is essential to mitigate the impacts of sarcopenia in cancer patients [8]. This individualized approach can significantly contribute to improving quality of life and clinical prognosis. Recent evidence also suggests that physical exercise can modulate biological systems involved in inflammation and muscle homeostasis, such as the purinergic system, suggesting new therapeutic perspectives [9].

Recent studies indicate that exercise can modulate pathways of the purinergic system, including extracellular nucleotide and adenosine signaling, CD39/CD73 activity, and P1 and P2 receptor activation, reducing inflammation and muscle catabolism [9]. Thus, supervised and individualized physical exercise programs may constitute a promising therapeutic strategy to mitigate the effects of sarcopenia in cancer patients, improving both quality of life and clinical prognosis [10].

Therefore, this narrative review aims to discuss the pathophysiological role of purinergic signaling in cancer-associated sarcopenia and to explore how physical exercise may modulate this system to reduce inflammation, preserve skeletal muscle mass, and improve quality of life in cancer patients. Literature sources were identified through searches in PubMed, Scopus, and Web of Science using combinations of terms related to exercise, inflammation, sarcopenia, skeletal muscle loss, and purinergic signaling. Articles were selected based on their scientific relevance, originality, methodological quality, and contribution to the topics addressed in this narrative review. Preference was given to recent publications and landmark studies in the field.

## Sarcopenia and cancer

Sarcopenia is characterized by the progressive loss of skeletal muscle mass, associated with reduced vitality and muscle strength. Although initially described as a condition related to aging, recent studies demonstrate that sarcopenia also has a high prevalence in cancer patients, affecting approximately 35.3% of the cancer population [11]. This condition is multifactorial, resulting from the interaction between metabolic changes that increase catabolism, systemic inflammation, and the adverse effects of antineoplastic treatments.

Clinically, sarcopenia is associated with negative outcomes. Sarcopenic patients exhibit greater chemotherapy-related toxicity, reduced time to tumor control, increased risk of postoperative complications, and increased mortality compared to those without the condition [12, 13]. Furthermore, this condition affects treatment response and increases susceptibility to nosocomial infections. Muscle mass loss also directly impacts quality of life, being associated with depressive symptoms and reduced functional capacity [14].

Early recognition of sarcopenia is essential for the implementation of timely therapeutic interventions, such as nutritional support with amino acid and vitamin supplements, combined with a multidisciplinary approach that includes resistance exercise programs, which have shown potential to reduce the effects of sarcopenia in cancer patients [15]. It is important to differentiate sarcopenia from conditions with similar manifestations, such as malnutrition and cachexia. Although all may present with muscle mass loss, they have distinct etiologies and require specific approaches. Malnutrition results from insufficient food intake or malabsorption, while cachexia is associated with inflammatory processes caused by underlying diseases, including cancer, and is often not reversible with nutritional support alone [16, 17]. Sarcopenia, in turn, can occur in isolation or in association with these conditions, and is specifically characterized by a reduction in muscle mass and function.

Sarcopenia is therefore a relevant clinical marker in cancer patients, not only contributing to muscle mass loss but also predicting clinical complications, functional impairment, and worse survival. International groups, such as the European Working Group on Sarcopenia in Older People (EWGSOP), the European Society for Clinical Nutrition and Metabolism Special Interest Group (ESPEN-SIG), and the International Working Group on Sarcopenia (IWGS), define criteria that encompass muscle mass loss, decreased strength, and reduced physical performance, highlighting the importance of early diagnosis [17, 18].

In summary, sarcopenia is a prevalent and clinically relevant condition in cancer patients, directly impacting prognosis, treatment tolerance, and quality of life. Early identification allows for the planning of nutritional and physical support strategies, essential for minimizing the adverse effects of muscle loss and improving clinical outcomes.

In this context, it becomes fundamental to understand the biological mechanisms that connect the inflammatory tumor environment to the progressive loss of muscle mass. Among these mechanisms, purinergic signaling stands out, activated by the exacerbated release of extracellular nucleotides resulting from cell damage, hypoxia, and persistent inflammation present in cancer. This pathway represents a pathophysiological link between the tumor microenvironment and the metabolic dysregulation of skeletal muscle, directly contributing to the development of cancer-associated sarcopenia.

## Purinergic system and sarcopenia

In cancer patients, sarcopenia is intimately linked to a systemic state of chronic inflammation, tissue hypoxia, and persistent cellular damage, characteristics that favor the release of ATP into the extracellular environment. In this condition, ATP ceases to act as an intracellular energy molecule and begins to exert a pro-inflammatory signaling function, activating purinergic receptors in muscle and immune cells. This phenomenon is known as one of the pillars of purinergic signaling in pathological contexts and has been associated with the metabolic dysregulation observed in inflammatory sarcopenia [9].

Extracellular ATP primarily activates receptors of the P2 family (P2X and P2Y), with emphasis on the P2 × 7 receptor, strongly related to inflammasome activation and the release of pro-inflammatory cytokines. In skeletal muscle, this sustained activation favors catabolic pathways, protein degradation, and impaired muscle homeostasis. In cancer patients, where ATP release is continuous due to the tumor microenvironment and permanent cellular stress, this pro-inflammatory signaling becomes chronic and directly contributes to muscle catabolism [19].

However, extracellular ATP is rapidly degraded by the ectonucleotidases CD39 and CD73, which convert ATP and ADP into AMP and, subsequently, into adenosine. This process, known as the CD39/CD73 axis, promotes a functional transition in purinergic signaling from an initially pro-inflammatory state mediated by P2 receptors to a predominantly immunosuppressive and metabolic modulating state mediated by P1 receptors activated by adenosine [20].

In cancer, overexpression of CD39 and CD73 in the tumor microenvironment has been described as an adenosine-mediated immune evasion mechanism, where extracellular ATP is degraded into adenosine that activates immunosuppressive pathways and dampens anti-tumor immunity [21]. Elevated adenosine levels can signal through P1 receptors such as A2A and A2B, which are known to modulate cellular metabolism and inflammatory responses in various tissues, suggesting that chronic systemic adenosinergic signaling may also interfere with myogenic differentiation, muscle regeneration, and the regulation of energy metabolism, thereby creating an unfavorable environment for maintaining muscle mass.

**Fig. 1:**
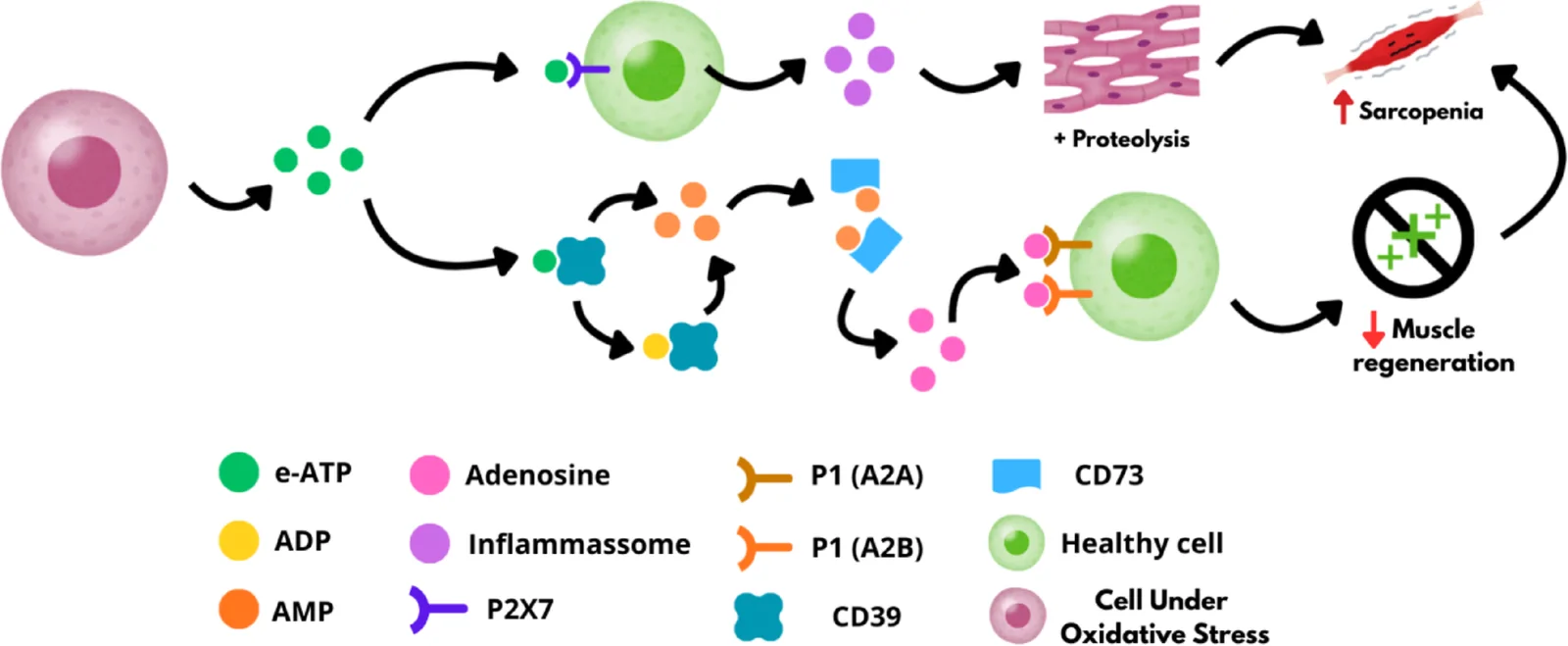
Purinergic system and sarcopenia

Therefore, the muscle of cancer patients is simultaneously exposed to two deleterious stimuli: the persistent activation of P2 receptors, which promotes inflammation and catabolism, and the chronic activation of P1 receptors, which favors immunosuppression and metabolic dysregulation. This purinergic dysregulation establishes a biochemical scenario conducive to the progression of cancer-associated sarcopenia, highlighting the purinergic system as a relevant pathophysiological axis that is still largely unexplored therapeutically [9]. Figure 1 illustrates purinergic signaling changes related to sarcopenia.

## Inflammation associated with the development of sarcopenia in patients with cancer

Aging is accompanied by a progressive increase in inflammatory cytokines, especially interleukin-6 (IL-6), which leads the body to a state of chronic low-grade inflammation, favoring the emergence of age-related frailties, including loss of muscle mass [22, 23].When this process is associated with cancer and oncological treatment, aging acts as a driver of the cachectic state, intensifying muscle wasting already stimulated by the systemic inflammatory environment [3]. Evidence indicates that inflammation is directly related to increased catabolism and reduced protein synthesis in skeletal muscle, being one of the main mechanisms involved in the development of sarcopenia [24].

This inflammation, however, is not only a consequence of cancer, but an integral part of the tumor microenvironment itself, often present even before the clinical manifestation of the neoplasm. This inflammatory environment favors tumor protection, prevents immunological recognition, and promotes damage to cellular DNA through the action of cytokines and chemokines, contributing to lesions that trigger malignant lesions [25, 26]. In cancer patients, the combination of chronic inflammation and malnutrition accelerates muscle depletion, which is consolidated as an important prognostic marker and a determining factor in therapeutic complications [27, 28]. It is estimated that about 20% of cancer cases are associated with organic inflammatory processes [29].

In parallel, tumor cells have a high energy demand and promote metabolic adaptations to sustain their rapid growth. Tumor hypoxia acidifies the microenvironment and stimulates angiogenesis, while the increased need for amino acids leads to the expression of cellular transporters, favoring the consumption of energy substrates from the host [30, 31]. In this context, proteins such as myostatin are increased in human neoplasms and act by inhibiting myoblast activation, blocking muscle hyperplasia and hypertrophy, directly contributing to the loss of muscle mass observed in these patients [32–34].

The systemic inflammatory state sustained by the tumor also promotes increased synthesis of MCP-1, which interacts with CCL-2 receptors in adipocytes and myocytes and stimulates myostatin expression. These mediators inhibit AMP-activated protein kinase (AMPK), a central regulator of cellular energy metabolism, responsible for balancing anabolic and catabolic pathways according to ATP availability. Suppression of AMPK favors insulin resistance, metabolic disorganization, and intensification of muscle protein manipulation, typical characteristics of cancer-associated sarcopenia and tumor cachexia syndrome [9, 35].

In this scenario, the exacerbated release of ATP and adenosine in the tumor microenvironment and peripheral tissues introduces purinergic signaling as an additional link between inflammation, metabolism, and muscle loss. Activation of the P2 × 7 receptor by high concentrations of ATP stimulates the production of pro-inflammatory cytokines through the NLRP3 inflammasome, while the release of ATP by the enzymes CD39 and CD73 increases the formation of adenosine, which exerts an immunosuppressive effect through the A2A and A2B receptors. This dual effect of systemic stimulation and local immunosuppression directly connects tumor progression to metabolic dysfunction and sarcopenia in cancer patients [36, 37].

## Role of the exercise in sarcopenia in patients with cancer

Physical exercise is currently recognized as one of the most effective non-pharmacological strategies for the prevention and treatment of sarcopenia in patients with cancer. Beyond its classical effects on muscle mass and strength, exercise exerts systemic actions on inflammatory, metabolic, and immunological pathways that are critically involved in cancer-associated muscle wasting [38, 39]. Accumulating evidence indicates that different exercise modalities—resistance, aerobic, combined, and functional training—can counteract the multifactorial mechanisms underlying sarcopenia, including chronic inflammation, metabolic dysregulation, and impaired muscle regeneration [40, 41].

### Exercise modalities and effects on muscle mass and function

Resistance exercise is the most consistently supported modality for increasing skeletal muscle mass and strength in oncological populations. By stimulating mechanical load–dependent signaling pathways, particularly the mTOR pathway, resistance training promotes muscle protein synthesis, attenuates proteolysis, and improves neuromuscular function [42]. Clinical trials demonstrate that resistance exercise, even at moderate intensities, leads to significant improvements in muscle strength, lean mass preservation, and physical performance in patients undergoing chemotherapy or radiotherapy [43, 44].

Aerobic exercise, although traditionally associated with cardiovascular adaptations, also contributes to the attenuation of sarcopenia by improving mitochondrial function, oxidative capacity, and metabolic flexibility in skeletal muscle [45]. Aerobic training reduces systemic inflammation, enhances insulin sensitivity, and improves oxygen delivery, thereby indirectly supporting muscle homeostasis (Roberts et al., 2013). Importantly, aerobic exercise has been shown to reduce cancer-related fatigue, one of the main barriers to physical activity in oncological populations [46].

Combined exercise programs that integrate resistance and aerobic training appear to offer synergistic benefits. These programs simultaneously target muscle hypertrophy, cardiorespiratory fitness, and metabolic health, resulting in superior improvements in functional capacity and quality of life compared to single-modality interventions [47]. Functional training further enhances balance, coordination, and autonomy, reducing fall risk and functional decline, particularly in frail or elderly cancer patients [48].

### Evidence for improvements in clinical outcomes and quality of life

Clinical and translational studies consistently report that structured exercise interventions increase muscle mass, muscular strength, and physical function in cancer patients with or at risk of sarcopenia [3, 39]. These adaptations are associated with improved treatment tolerance, reduced chemotherapy-related toxicity, shorter hospital stays, and enhanced overall survival [28]. In addition to musculoskeletal benefits, exercise improves cardiovascular and metabolic health, reduces fatigue, anxiety, and depressive symptoms, and significantly enhances health-related quality of life [49].

### IL-6 as a myokine versus pro-inflammatory cytokine: a purinergic perspective

Interleukin-6 (IL-6) plays a dual and context-dependent role in cancer-associated sarcopenia. In chronic inflammatory states driven by cancer, IL-6 acts as a pro-inflammatory cytokine, promoting muscle catabolism, insulin resistance, and activation of proteolytic pathways [24, 38]. Sustained elevations of IL-6 are strongly associated with poor prognosis, cachexia, and reduced muscle mass in cancer patients [50].

In contrast, during and after physical exercise, IL-6 is acutely released from contracting skeletal muscle and functions as a myokine with anti-inflammatory and metabolic regulatory properties. Exercise-induced IL-6 stimulates the production of anti-inflammatory cytokines, enhances lipid oxidation, improves glucose uptake, and supports muscle regeneration [51, 52].

This dichotomy is closely linked to purinergic signaling. Chronic tumor-driven inflammation is associated with excessive extracellular ATP release and sustained activation of P2 receptors, particularly P2 × 7, which amplifies IL-6 production and inflammasome activation [9, 36]. In contrast, exercise-induced ATP release is transient and tightly regulated, favoring adaptive purinergic signaling that supports metabolic homeostasis and anti-inflammatory responses [53].

### Exercise as an immunometabolism modulator

Exercise should be regarded as an immunometabolism modulator rather than solely a muscle-targeted intervention. By influencing purinergic signaling, exercise modulates immune cell function, cytokine production, and energy metabolism across multiple tissues [54, 55]. Exercise regulates the activity of the ectonucleotidases CD39 and CD73, promoting the controlled conversion of extracellular ATP into adenosine. This shift reduces pro-inflammatory P2 receptor signaling while enhancing adenosine-mediated P1 receptor activation, which is associated with anti-inflammatory, cytoprotective, and immunoregulatory effects [20, 37].

Through these mechanisms, exercise attenuates systemic inflammation, limits muscle catabolism, and improves metabolic efficiency, creating a physiological environment that counteracts the progression of cancer-associated sarcopenia [39, 41].

## Modulation of the purinergic system by exercise in cancer patients

In cancer, sarcopenia is associated with a systemic environment characterized by persistent inflammation, metabolic stress, and activation of catabolic pathways in skeletal muscle. In this scenario, the purinergic system plays a central role in mediating cell communication through extracellular nucleotides and nucleosides, especially ATP and adenosine. Alterations in this signaling are directed towards the progression of muscle wasting, making the purinergic system a relevant axis for interventions capable of modulating these processes [36, 55].

The excessive and sustained release of ATP into the extracellular environment is a characteristic of the tumor microenvironment and tissues subjected to continuous damage. In cancer patients, extracellular ATP predominantly acts as a pro-inflammatory signal by activating P2 purinergic receptors, more specifically P2 × 7, triggering the production of inflammatory cytokines such as IL-6 and IL-1β, increased oxidative stress, and the activation of proteolytic pathways in skeletal muscle. Thus, these mechanisms directly stimulate muscle catabolism and the development of cancer-associated sarcopenia [38, 56].

Physical exercise directly interferes with purinergic signaling by modulating the pattern of extracellular ATP release. In general, exercise promotes a transient and controlled release of ATP, associated with adaptive responses such as modulation of P2 receptors and ectonucleotidases, contributing to systemic anti-inflammatory and metabolic benefits [57]. In conditions of chronic disease, such as chronic kidney disease, exercise has been shown to specifically regulate P2 × 7 receptor activity and purinergic signaling in skeletal muscle, attenuating catabolic pathways and improving muscle function [53]. Furthermore, physical exercise can influence the activity of the ectonucleotidases CD39 and CD73, responsible for the sequential conversion of ATP to adenosine in the extracellular space. The regulation of this axis favors the balance between ATP and adenosine, promoting a controlled increase in adenosine-mediated signaling. Activation of P1 purinergic receptors is associated with anti-inflammatory, immunomodulatory, and cytoprotective effects, contributing to the attenuation of catabolic processes in skeletal muscle [54, 58, 59].

**Fig. 2:**
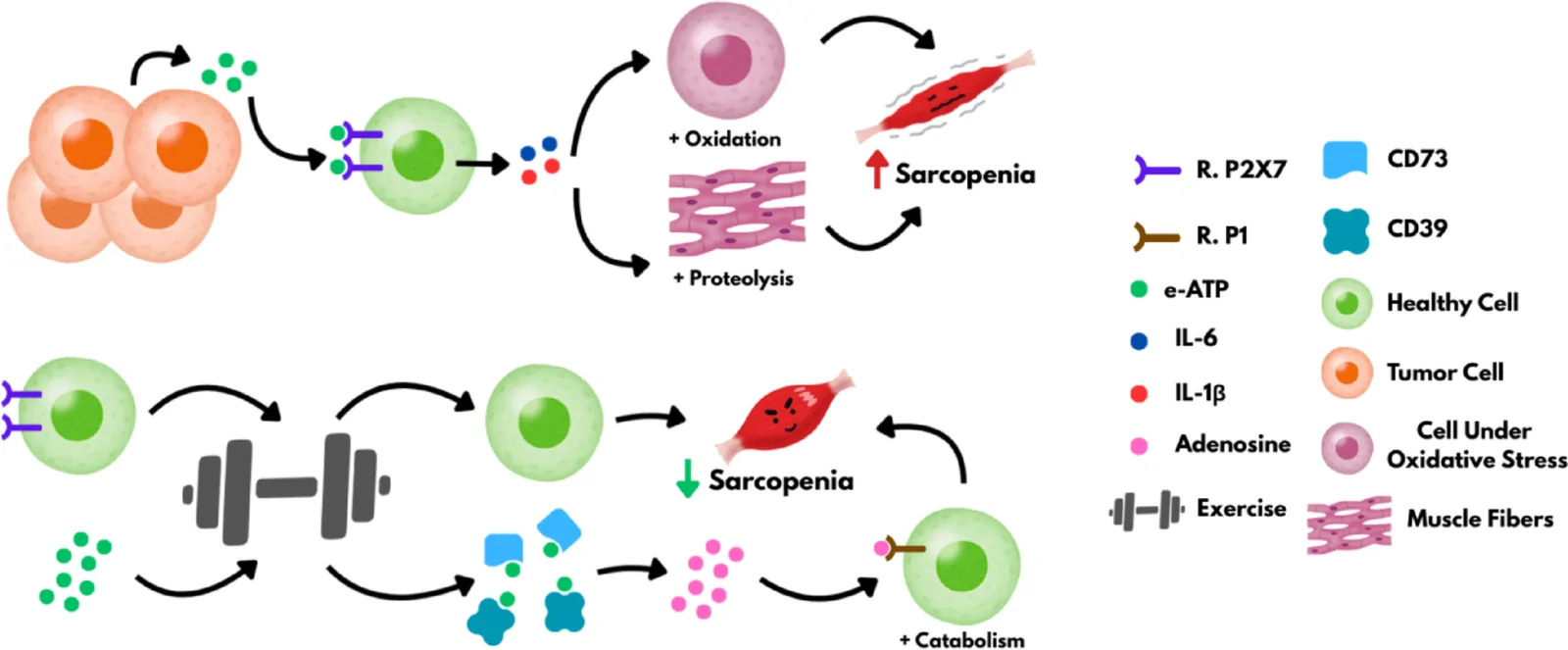
Modulation of the purinergic system by exercise in cancer patients

Thus, by promoting a shift from pro-inflammatory purinergic signaling mediated by ATP and P2 receptors to a profile regulated by adenosine and P1 receptors, physical exercise directly acts on molecular mechanisms involved in cancer-associated sarcopenia. Experimental and clinical evidence indicates that this modulation is associated with reduced systemic hypertension, decreased muscle catabolism, and preservation of muscle mass and function, reinforcing exercise as a promising non-pharmacological strategy in the management of this condition [39, 60]. Figure 2 shows the modulation of purinergic signalling during exercise.

## Future proposals

### Exercise as a Targeted anti-inflammatory and immunometabolism intervention

Future research and clinical strategies should position supervised and individualized exercise programs as a central non-pharmacological intervention to counteract cancer-associated sarcopenia through modulation of chronic inflammation. Given that persistent low-grade inflammation represents a hallmark of both cancer progression and sarcopenia, exercise prescriptions must be tailored according to the patient’s inflammatory burden, metabolic status, and treatment-related toxicities [24, 39]. Supervised exercise programs delivered by multidisciplinary teams are essential to ensure safety and adherence, while enabling controlled modulation of inflammatory pathways that contribute to muscle catabolism.

From a translational perspective, exercise should be viewed as a biological stimulus capable of reprogramming inflammatory signaling rather than merely a supportive rehabilitation strategy. Individualized exercise interventions may attenuate systemic cytokine production, reduce inflammasome activation, and restore immunometabolism homeostasis, thereby directly addressing mechanisms central to inflammation-driven sarcopenia.

### Exercise Combined with conventional anti-cancer therapies: an inflammatory perspective

The integration of exercise with conventional cancer therapies, including chemotherapy and immunotherapy, represents a promising avenue to mitigate therapy-induced inflammation and muscle wasting. Anticancer treatments frequently exacerbate systemic inflammatory responses, contributing to immune dysfunction and metabolic imbalance. Emerging evidence suggests that exercise may counteract these effects by reducing pro-inflammatory cytokine release and improving immune regulation [28, 49].

Importantly, exercise-induced modulation of purinergic signaling—via regulation of extracellular ATP availability and ectonucleotidase activity—may influence immune checkpoint pathways and inflammatory signaling cascades relevant to tumor progression and host response [36, 58]. These interactions warrant further investigation, particularly in the context of immunotherapy, where inflammation and immune exhaustion play decisive roles in treatment efficacy.

### Exercise prescription to modulate inflammatory load: intensity, frequency, and safety

Defining optimal exercise intensity, frequency, and volume remains a key challenge for targeting inflammation in cancer patients. While moderate-intensity resistance and aerobic exercise are generally safe, excessive training loads may exacerbate systemic stress and inflammatory responses in vulnerable individuals. Therefore, exercise prescriptions should adopt adaptive and progressive models, guided by continuous monitoring of inflammatory markers, fatigue levels, and clinical status [46].

From the standpoint of inflammatory biology, future guidelines should aim to identify exercise “doses” that effectively suppress chronic inflammation and catabolic signaling while preserving immune competence. Safety considerations are particularly relevant in patients with immune suppression, anemia, cardiotoxicity, or bone metastases, underscoring the importance of personalized and mechanistically informed exercise prescriptions.

### Knowledge gaps and research priorities in inflammation and purinergic signaling

Despite growing interest in exercise as an anti-inflammatory intervention, critical gaps remain regarding its specific effects on purinergic signaling in cancer-associated sarcopenia. There is a clear need for randomized controlled trials explicitly designed to evaluate how different exercise modalities influence extracellular ATP release, P2 × 7 receptor activation, CD39/CD73 expression, and adenosine-mediated anti-inflammatory signaling in cancer patients [54, 55].

Furthermore, the exploration of purinergic biomarkers—such as circulating ATP and adenosine concentrations, ectonucleotidase activity, and purinergic receptor expression—may provide valuable tools to monitor inflammatory responses to exercise interventions [9]. Integrating these biomarkers with classical inflammatory markers, including IL-6, TNF-α, and CRP, could support the development of precision exercise-based strategies aimed at suppressing inflammation-driven muscle wasting.

Future studies should also investigate personalized exercise interventions based on inflammatory and purinergic profiles, advancing the concept of exercise as a targeted anti-inflammatory therapy. Such an approach aligns closely with the scope of *Inflammation Research*, emphasizing mechanistic insight, translational relevance, and the modulation of inflammatory pathways as central outcomes.

### Exercise, immunometabolism, inflammation, and prevention of skeletal muscle loss

Physical exercise is widely recognized as one of the most effective non-pharmacological strategies for preventing sarcopenia. Beyond its role in preserving skeletal muscle mass and function, exercise exerts profound effects on immunometabolic regulation, an emerging concept describing the interplay between immune responses and cellular metabolism. Sarcopenia is increasingly viewed as a multifactorial condition driven not only by aging and physical inactivity but also by chronic low-grade inflammation, mitochondrial dysfunction, and metabolic imbalance. Elevated circulating levels of pro-inflammatory cytokines, including tumor necrosis factor-alpha (TNF-α) and interleukin-6 (IL-6), promote protein degradation, anabolic resistance, and impaired muscle regeneration. Regular physical exercise counteracts these alterations through the release of myokines, enhancement of mitochondrial biogenesis, improvement of oxidative metabolism, and attenuation of chronic systemic inflammation, thereby promoting muscle homeostasis and metabolic resilience [51, 61, 62].

Among the different exercise modalities, resistance training is consistently recognized as the most effective intervention for preserving and increasing skeletal muscle mass. Its benefits are mediated by the activation of anabolic signaling pathways, particularly the insulin-like growth factor-1 (IGF-1)/AKT/mechanistic target of rapamycin (mTOR) axis, which stimulates muscle protein synthesis while suppressing proteolytic pathways. Resistance exercise also promotes favorable immunometabolic adaptations by improving glucose utilization, enhancing mitochondrial efficiency, and reducing inflammatory signaling associated with muscle catabolism. Furthermore, resistance training stimulates satellite cell activation and muscle regeneration, reinforcing its central role in the prevention and treatment of sarcopenia [63, 64].

Although aerobic exercise is less effective in inducing muscle hypertrophy, it plays a fundamental role in metabolic and inflammatory regulation. Aerobic training enhances mitochondrial biogenesis through activation of peroxisome proliferator-activated receptor gamma coactivator-1 alpha (PGC-1α), improves insulin sensitivity, increases oxidative phosphorylation, and contributes to systemic anti-inflammatory effects. These adaptations are particularly relevant in individuals with chronic diseases and cancer, in whom persistent inflammation and metabolic disturbances accelerate muscle wasting. Additionally, aerobic exercise promotes the release of exercise-induced signaling molecules, known as exerkines, which participate in the crosstalk between skeletal muscle, immune cells, and metabolic tissues, contributing to whole-body metabolic homeostasis [65, 66].

Recent evidence suggests that combined exercise programs integrating resistance and aerobic training may provide the greatest overall benefits for individuals at risk of sarcopenia. By simultaneously targeting anabolic signaling, mitochondrial metabolism, inflammatory regulation, and physical performance, these interventions improve muscle strength, preserve lean mass, enhance cardiorespiratory fitness, and improve quality of life. Consequently, combined exercise programs represent a comprehensive strategy capable of counteracting the multifactorial mechanisms underlying skeletal muscle loss. Nevertheless, resistance training remains the most effective modality for directly preserving and increasing skeletal muscle mass [67].

Beyond its established effects on inflammation and muscle metabolism, exercise may also influence the purinergic system, which has emerged as a critical regulator of immunometabolic homeostasis. During muscle contraction, extracellular adenosine triphosphate (ATP) is released into the interstitial environment and acts as a signaling molecule involved in immune cell activation, tissue remodeling, mitochondrial adaptation, and skeletal muscle regeneration. Extracellular ATP functions as a danger-associated molecular pattern (DAMP), triggering transient inflammatory responses that are essential for tissue repair and adaptation to exercise [55, 68).

Subsequently, extracellular ATP is hydrolyzed by the ectonucleotidases CD39 and CD73, generating adenosine, a potent immunoregulatory metabolite. Through activation of adenosine receptors, particularly A2A and A2B receptors, adenosine limits excessive inflammatory responses, promotes tissue recovery, and contributes to the restoration of immune homeostasis. This ATP–adenosine balance constitutes a central immunometabolic checkpoint linking cellular energy status to immune regulation, inflammation resolution, and metabolic adaptation [68, 69].

Recent studies suggest that exercise-induced modulation of purinergic signaling contributes to skeletal muscle preservation by regulating macrophage polarization, mitochondrial function, inflammatory responses, and regenerative processes. In particular, purinergic receptors have been implicated in muscle repair and regeneration, highlighting the importance of extracellular nucleotide signaling in maintaining muscle integrity during aging and chronic disease [70]. Moreover, considering the recognized role of the CD39/CD73–adenosine axis in cancer-associated immunosuppression and cachexia, modulation of purinergic signaling may represent an important mechanistic link between exercise, inflammation control, muscle maintenance, and cancer-related muscle wasting. Therefore, the ATP–adenosine pathway may constitute an additional immunometabolic mechanism underlying the beneficial effects of exercise on skeletal muscle preservation, functional capacity, and inflammatory regulation [69].

## Conclusions

Accordingly, this review highlights that sarcopenia, frequently observed in patients with cancer, arises from a complex interplay between persistent systemic inflammation, metabolic stress, and the activation of catabolic pathways in skeletal muscle, in which purinergic signaling plays a central role. Exacerbated release of extracellular ATP within the tumor microenvironment and peripheral tissues, followed by sustained activation of purinergic receptors, contributes substantially to the disruption of muscle homeostasis, thereby accelerating the progression of muscle mass and functional decline in this population.

Within this inflammatory context, physical exercise emerges as a relevant non-pharmacological strategy capable of modulating purinergic signaling and attenuating the inflammatory and catabolic processes associated with cancer-related sarcopenia. Current evidence indicates that regular exercise promotes a controlled and transient release of extracellular ATP, limiting chronic P2 receptor activation while favoring a more balanced purinergic profile. This shift enhances adenosine-mediated P1 receptor signaling, which is associated with anti-inflammatory, cytoprotective, and immunoregulatory effects. Consequently, exercise acts not only at the level of skeletal muscle but also as a systemic immunometabolism modulator.

Combined resistance and aerobic exercise programs appear particularly effective in preserving muscle mass and improving physical function in patients with cancer. Resistance training primarily stimulates muscle protein synthesis and strength maintenance, whereas aerobic exercise improves cardiorespiratory fitness, metabolic health, and the regulation of systemic inflammation. When integrated, these exercise modalities may potentiate favorable adaptations in purinergic signaling, promoting coordinated improvements in energy metabolism and inflammatory control.

Therefore, the implementation of supervised and individualized exercise programs represents a promising strategy for the management of sarcopenia in cancer patients, with positive implications for physical function, quality of life, and potentially clinical prognosis. Nevertheless, despite significant advances, further clinical studies are required to elucidate the specific purinergic mechanisms underlying exercise-induced benefits and to enable the personalization of exercise interventions based on individual inflammatory, metabolic, and functional profiles.

## Data Availability

Not applicable.
